# Robust Vehicle Detection under Various Environments to Realize Road Traffic Flow Surveillance Using an Infrared Thermal Camera

**DOI:** 10.1155/2015/947272

**Published:** 2015-02-11

**Authors:** Yoichiro Iwasaki, Masato Misumi, Toshiyuki Nakamiya

**Affiliations:** ^1^Faculty of Industrial and Welfare Engineering, Tokai University, 9-1-1 Toroku, Higashi-ku, Kumamoto 862-8652, Japan; ^2^Freelance Image Processing Engineer, 228-602 Ueki, Ueki-machi, Kita-ku, Kumamoto 861-0132, Japan

## Abstract

To realize road traffic flow surveillance under various environments which contain poor visibility conditions, we have already proposed two vehicle detection methods using thermal images taken with an infrared thermal camera. The first method uses pattern recognition for the windshields and their surroundings to detect vehicles. However, the first method decreases the vehicle detection accuracy in winter season. To maintain high vehicle detection accuracy in all seasons, we developed the second method. The second method uses tires' thermal energy reflection areas on a road as the detection targets. The second method did not achieve high detection accuracy for vehicles on left-hand and right-hand lanes except for two center-lanes. Therefore, we have developed a new method based on the second method to increase the vehicle detection accuracy. This paper proposes the new method and shows that the detection accuracy for vehicles on all lanes is 92.1%. Therefore, by combining the first method and the new method, high vehicle detection accuracies are maintained under various environments, and road traffic flow surveillance can be realized.

## 1. Introduction

Traffic image sensors can measure vehicle positions and vehicle movements on multilane. By using traffic image sensors, we can expect to realize automatic traffic flow surveillance to find traffic incidents without time delay. Many researches on vehicle detection using visible-light image sensors have been proposed [1–4]. However, visible-light image sensors have some defects which are described below.

Vehicle cast shadows impair vehicle detection. To remedy this problem, complicated vehicle cast shadow elimination methods have been proposed [5, 6]. Shadows cast by buildings and other landmarks also decrease vehicle detection accuracy. Moreover, it is difficult to detect vehicles under poor visibility conditions such as thick fog and heavy rain. In traffic flow surveillance, it is very important to detect vehicles with high accuracy around the clock regardless of changing environments.

In recent years, we have been developing the vehicle detection methods robust for various environments using the physical characteristics of an infrared thermal camera. We have proposed a vehicle detection method using Viola-Jones detector to detect the windshields and their surroundings of vehicles [7]. We have developed a vehicle detection method based on the method proposed in [7] and proved in experimental results that high vehicle detection accuracies are maintained in several environments which include poor visibility condition of snow and thick fog [8]. Reference [9] describes that the highest attenuation occurs in thick fog as compared with other poor visibility conditions. Even in the snow and thick fog condition of the highest attenuation, the vehicle detection method using Viola-Jones detector [8] (henceforth referred to as “method 1”) can detect vehicles. The vehicle detection method using Viola-Jones detector decreases its detection accuracy in a condition in winter season. To remedy this problem, we have developed another vehicle detection method without relying on Viola-Jones detector [10]. This method detects tires' thermal energy reflection areas as the targets of vehicles and is effective in winter season. In this way, we have been developing our original vehicle detection methods.

The method proposed in [10] did not achieve high detection accuracy for vehicles on left-hand and right-hand lanes except for two center-lanes. Therefore, we must develop a new method (henceforth referred to as “method 2”) based on the method proposed in [10] to increase the detection accuracy for vehicles on all lanes.

This paper proposes method 2 and shows that the detection accuracy for vehicles on all lanes is over 90%. Therefore, by combining method 1 and method 2, high vehicle detection accuracies are maintained under various environments. One of the vehicle detection accuracy standards decided by the national police agency of Japan requires over 90% accuracy.

A few vehicle detection methods using infrared thermal images have been proposed [11, 12]. However, these methods do not show that vehicles in heavy traffic can be detected. Therefore, these methods are not useful for traffic flow surveillance. We will compare the proposed methods with these two state-of-the-art algorithms [11, 12] in Section 3.4.

We also described in [10] the advantages of an infrared thermal camera as a passive sensor compared with a millimeter wave radar as an active sensor. A millimeter wave radar has some weaknesses such as interference and unsatisfied spatial resolution [13–17].

First, we will briefly explain method 1, the experiments, and their results in Section 2. Second, we will propose method 2 and will explain the experiments, their results, and the combination of method 1 and method 2 in Section 3. Third, we will show an automatic traffic flow surveillance using method 1 and method 2 in Section 4. Finally, we will present our conclusions in Section 5.

## 2. Method 1, the Experiments, and Their Results [8]

**Figure 1:**
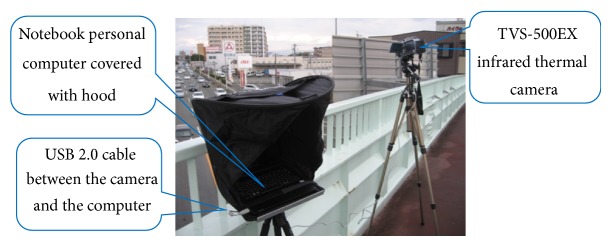
The infrared thermal camera and the notebook personal computer covered with the hood on a pedestrian bridge.

**Figure 2:**
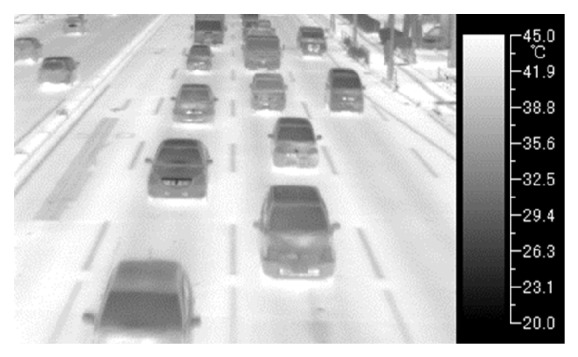
A frame of obtained thermal images.

Figure 1 shows TVS-500EX [18] infrared thermal camera and the notebook personal computer to capture thermal images on a pedestrian bridge.  Figure 2 shows one frame of obtained thermal images. The frame size is 320 × 240 pixels, and each pixel has 256 gray levels. The frame rate is 60 fps. We also use TVS-200 [19] infrared thermal camera. Two cameras have the same physical property.

### 2.1. The Algorithms of Method 1

Method 1 consists of three algorithms: spatiotemporal image processing, vehicle pattern recognition using the Viola-Jones detector [20, 21], and correction procedures for misrecognition of vehicles.

**Figure 3:**
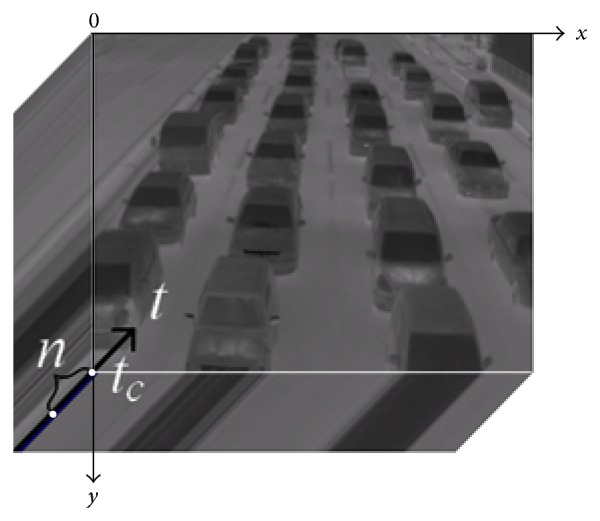
An example of spatiotemporal images.

The spatiotemporal image processing detects the regions of moving vehicles based on the standard deviation of each pixel value calculated from *n* frames in the past.  Figure 3 shows an example of spatiotemporal images. In Figure 3, *x* and *y* are space-axes, *t* is time-axis, and *t*_*c*_ is the current time. The pixel values in some areas are changed by movements of vehicles in *n* frames (*n* = 30 in our experiments). On the other hand, other pixel values in the background or stopped vehicles are affected by random noise, but the pixel values are nearly unchanged. Therefore, we can distinguish between the areas of moving vehicles and those of the background or stopped vehicles based on the standard deviations. The spatiotemporal image processing is also used in method 2.

The vehicle pattern recognition using the Viola-Jones detector detects the windshield and its surroundings as the target of pattern recognition. By using the upper part of a vehicle like the windshield and its surroundings as the target of pattern recognition, we can realize robust vehicle detection even when they are stopped one after another with a short vehicular gap. We have conducted training with positive and negative sample images to obtain a multistage cascade of classifiers and finally managed pattern recognition of vehicles with the obtained multistage cascade of classifiers.

The correction procedures for misrecognition of vehicles are applied to vehicle pattern recognition results to increase vehicle detection accuracy. When the omission of vehicle detection occurs in the vehicle pattern recognition, the omitted vehicle position is searched from the vehicle position in the previous frame by using pattern matching. The size of windshield is bigger as the vehicle comes nearer to an infrared thermal camera. We obtained the regression equation by a regression analysis on the relationship between *y*-positions and the sizes of the recognition-target areas. The obtained regression equation will be shown in Section 3.2 as (3). The size of the recognition-target area is calculated by the obtained regression equation after substitution of *y*-position. If the size of detected object is less than 40% of the calculated size, the detected object is treated as a nonrecognition target. If two rectangles are overlapped on a vehicle, the smaller rectangle is deleted.

By combining the two kinds of processing (the spatiotemporal image processing, and the vehicle pattern recognition with the correction procedures) in the same image, the position of each vehicle is specified, and its movement is also classified. The speed of each vehicle can be classified based on the ratio of the area of a moving vehicle in a rectangle which shows the windshield and its surroundings of a vehicle. The ratio of the area of a moving vehicle in a rectangle is increased as the vehicle speed is increased. In proposed method, we have classified three movement categories: stopped vehicle, slow moving vehicle, and fast moving vehicle.

### 2.2. Vehicle Detection Experiments

**Figure 4:**
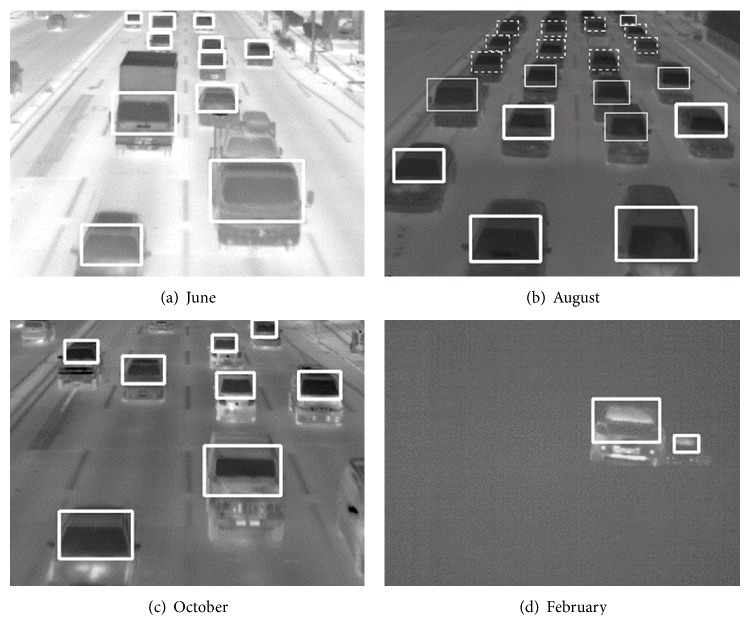
Some vehicle detection results by method 1.

Figure 4 shows some vehicle detection results in June, August, October, and February. The dotted lines, the thin lines, and the bold lines in Figure 4 show three movement categories of vehicles: stopped vehicles, slow moving vehicles, and fast moving vehicles, respectively.  Table 1 shows vehicle detection results using three video clips taken in June, August, and October.

The detection accuracy in Table 1 is defined as

(1) $$\begin{array}{c} A=\frac{n_{1}}{n_{2}}\cdot 100\left(\%\right), \end{array}$$

where *A* is the detection accuracy, *n*_1_ is the number of vehicles detected by proposed method, and *n*_2_ is the number of vehicles counted by sight. The false detection in Table 1 means detection of two locations on a vehicle or detection of nonrecognition targets.

Method 1 also detected vehicles robustly in 222 continuous images with 1/60 s interval in a video clip taken under a snow and thick fog condition in a winter cold day.  Figure 4(d) shows an image of the detected 222 continuous images. The total measurement time in February is short compared with the measurement time in the other three months because the visibility was very low, the road surface was frozen, and it was dangerous to stay at the shooting location for long minutes.

The temperature on windshields is usually lower than that in their exterior in spring, summer, and autumn as shown in Figures 4(a)–4(c). On the other hand, the temperature on windshields is higher than that in their exterior in winter cold days as shown in Figure 4(d). Therefore, the relationship between the two temperatures is reversed in winter cold days. Method 1 can detect vehicles in winter cold days by using the negative transformation of the original thermal images. We have proved that this preprocess is effectively useful for vehicle detection in a winter cold day of snowy and deep foggy weather. The negative transformation can be obtained simply by

(2) $$\begin{array}{c} g=I_{\max}-f, \end{array}$$

where *g* is transformed pixel value, *I*_max⁡_ is maximum pixel value (*I*_max⁡_ = 255 in our experiments), and *f* is input pixel value. In such cases, method 1 can work by simply employing the negative transformation for the original images as a preprocessing.

To survey road traffic flow, it is important to measure the position and movement of each vehicle on a road with high accuracy under various environments. There is no need to obtain the accurate speed of each vehicle. A rough estimate for vehicle movements suffices for road traffic surveillance. Therefore, method 1 and method 2 which will be described in Section 3 categorize roughly three movements.

## 3. Method 2, the Experiments, Their Results, and the Combination of Method 1 and Method 2

### 3.1. The Necessity of Method 2

**Figure 5:**
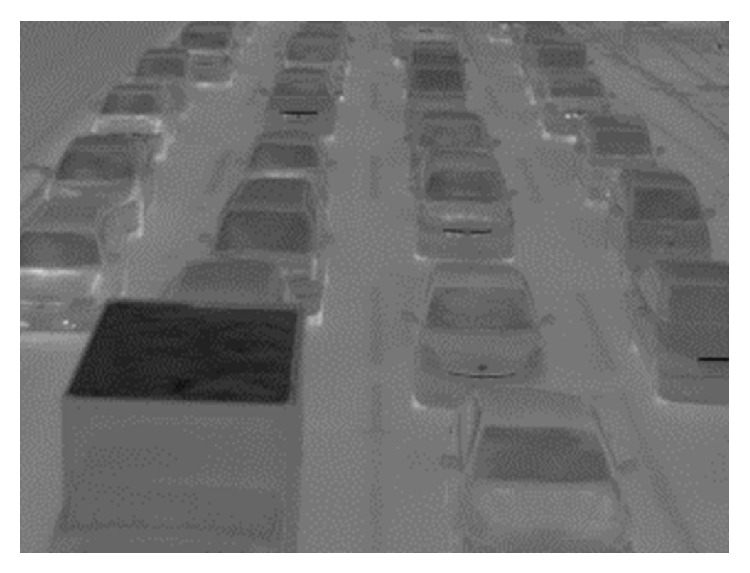
A thermal image in which the temperatures of the windshields and those of the exterior of the windshields are similar.

In a condition in winter season, the vehicle detection accuracy of method 1 decreases because the difference between two temperatures, the temperature of the windshield and the temperature of the exterior of the windshield, is reduced as shown in Figure 5. Therefore, it is necessary to realize method 2 without relying on the pattern recognition. By combining method 1 and method 2, high vehicle detection accuracies will be realized under various environments.

### 3.2. A Proposed Algorithm of Method 2

An infrared thermal camera receives mainly two types of thermal energy in traffic flow surveillance: the thermal emission energy received directly from vehicles and the vehicles' thermal energy reflected by a road surface. We have found out that the thermal images taken in winter season offer high pixel values in the tires' thermal energy reflection areas on a road surface. When the open air temperature is low like in winter season, the temperature difference between tires and a road surface becomes high [22]. Therefore, we can find high pixel values in the tires' thermal energy reflection areas just in winter season. If the pixel values located on both sides of vehicles are automatically measured, the vehicles can be detected. The detection accuracy of the method proposed in [10] maintains over 90% accuracy for vehicles on two center-lanes. However, the detection accuracy for vehicles on left-hand and right-hand lanes is decreased because the mean of temperature on each lane is different and the shapes of tires' thermal energy reflection areas are also different from those of two center-lanes.

To increase the detection accuracy for vehicles on all lanes, it is necessary to detect exactly the tires' thermal energy reflection areas in each lane. Japan uses left-hand traffic, and the left-hand lane nearest the road shoulder is called lane 1. The lanes are numbered from left-hand lane to right-hand lane as shown in Figure 9. We have improved mainly the algorithm proposed in [10] on the following two points.

1. In lanes 1 and 2, the right side of vehicles is the detection area. In lanes 3 and 4, the left side of vehicles is the detection area. The edges of the detected side of vehicles must be extracted accurately.
2. The mean of temperature on each lane is different because traffic volume and occupancy in each lane are different. The tires' thermal energy reflection areas must be emphasized effectively from the road surface without being influenced by the temperature differences of lanes.

By improving the algorithm, we have developed method 2 and achieved the accuracy over 90% for vehicles in all lanes.

**Figure 6:**
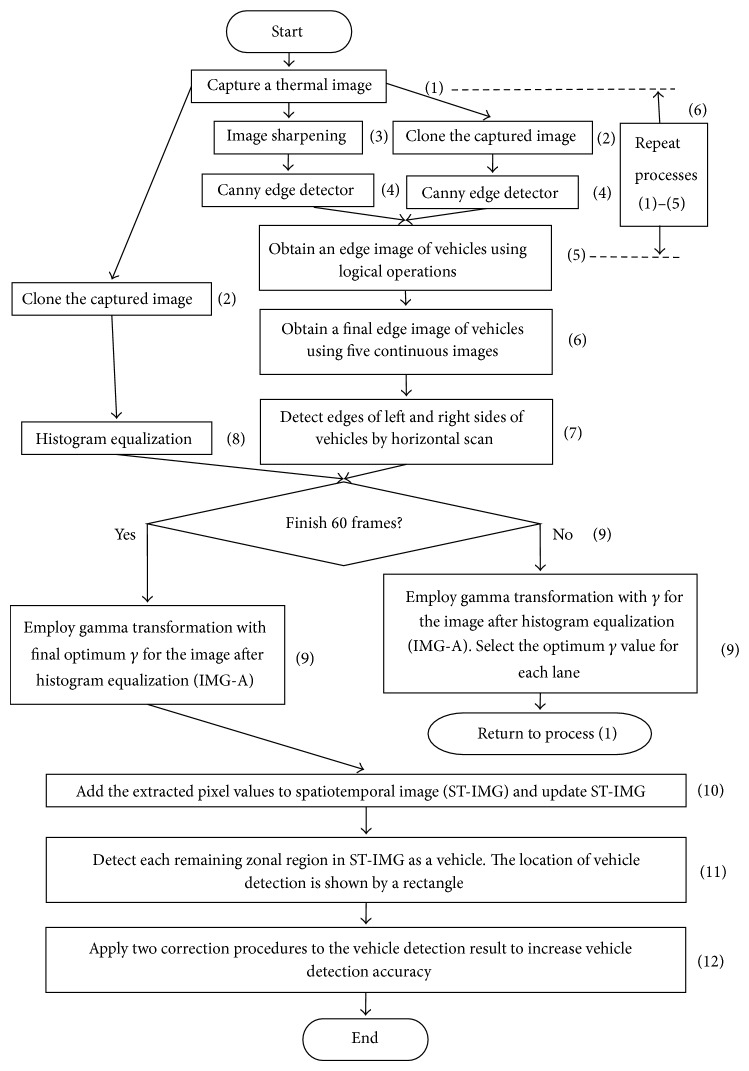
The flowchart of method 2.

We describe the algorithm of method 2 by use of Figure 6. Each number as shown below corresponds to that in Figure 6.

**Figure 7:**
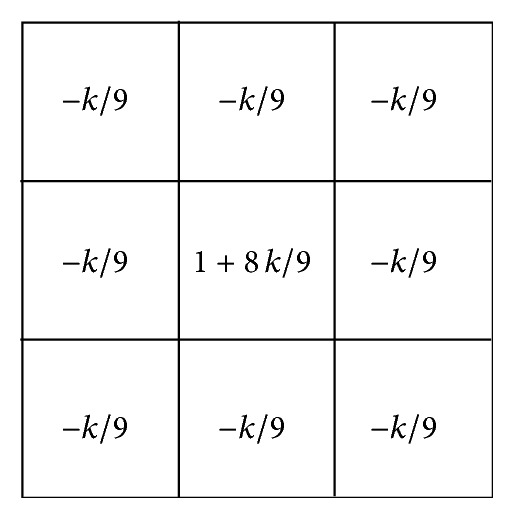
The kernel for unsharp masking.

**Figure 8:**
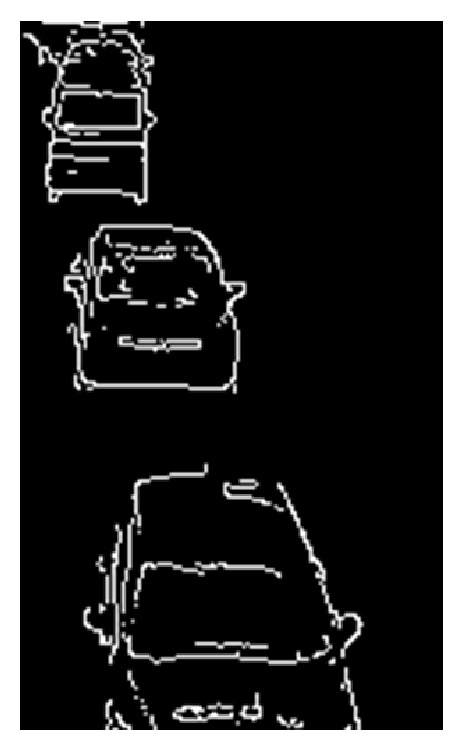
An obtained edge image in lane 2.

**Figure 9:**
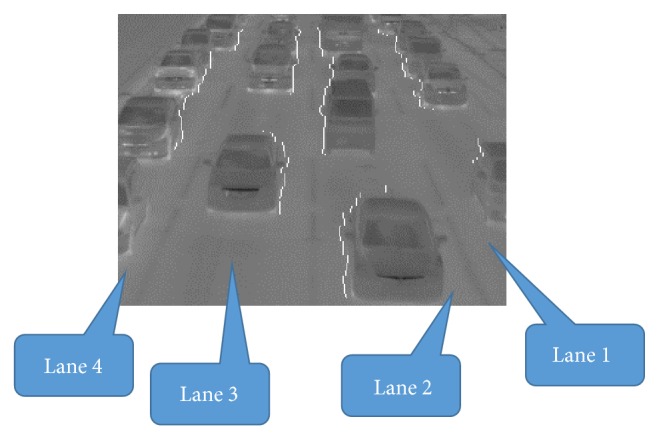
Detected edges of left and right sides of vehicles.

**Figure 10:**
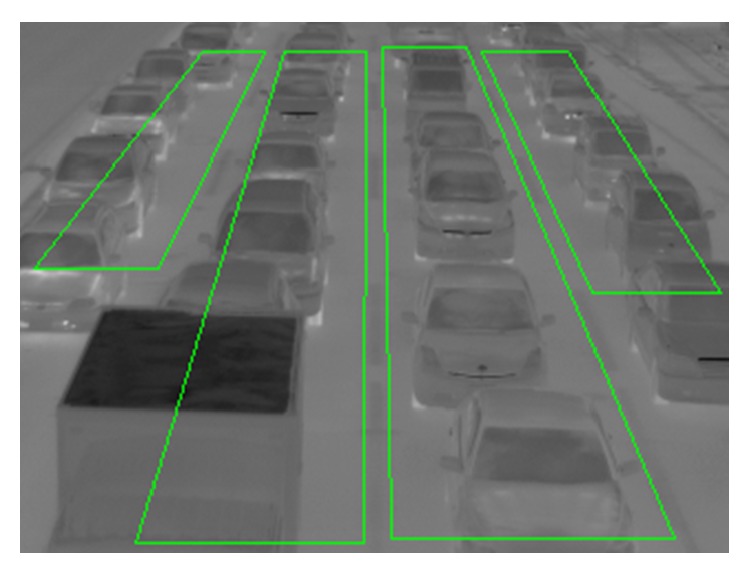
An example of four ROIs which are indicated in green lines.

**Figure 11:**
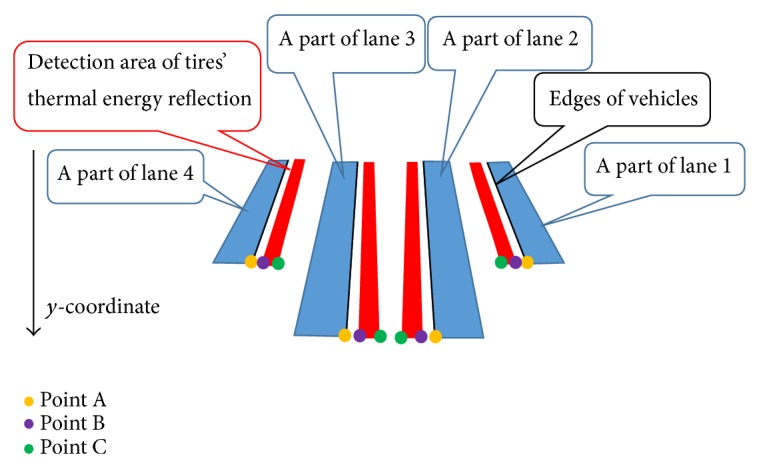
Positions of edges of vehicles and detection areas.

**Figure 12:**
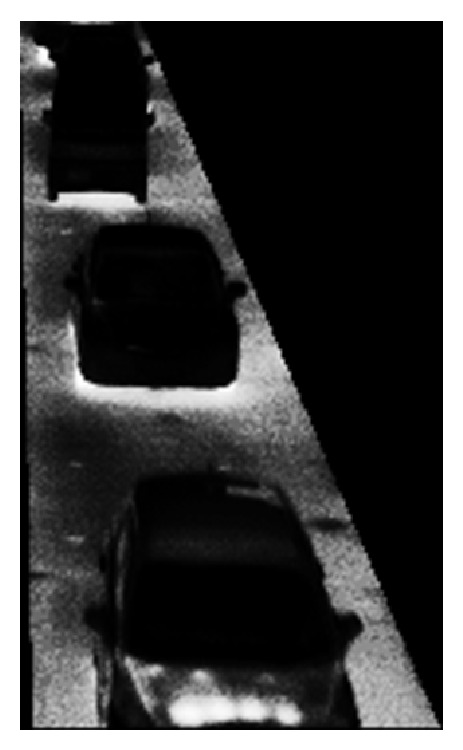
An image in lane 2 after final optimum gamma transformation.

**Figure 13:**
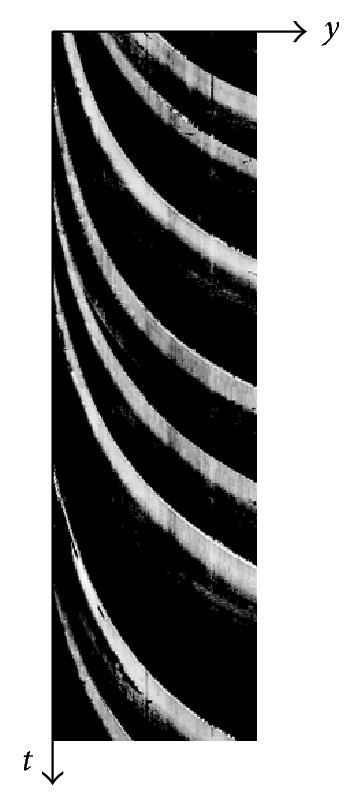
A spatiotemporal image (ST-IMG) in lane 2.

**Figure 14:**
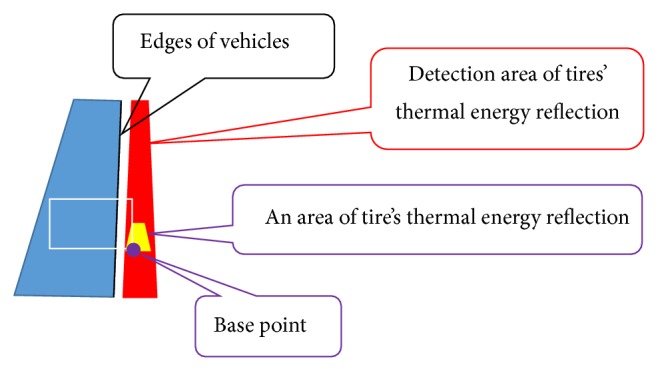
The position of the rectangle which shows a detected vehicle.

(1) A thermal image is captured.

(2) The captured image is cloned, and three same images are obtained. This is to do three kinds of image processing for the original image.

(3) Image sharpening using the unsharp masking with *k* shown in Figure 7 is employed for the original image. The variable *k* in Figure 7 is a weight. The *k* is greater than or equal to 0 for generality. The degree of sharpening is increased as the value of *k* is higher.

(4) Canny edge detector with two thresholds, *T*_*L*_ and *T*_*H*_, is employed for sharpened image and original image.

(5) Two kinds of edge images are obtained. The edges of vehicles in the edge original image are broken. The edges of vehicles in the edge sharpened image clearly appear, but the image contains random noise. To remove the random noise, logical operation AND is employed between two continuous edge sharpened images. After the logical operation, connected components are searched by use of the remained edge pixels. Logical operation OR is employed between the obtained image which contains connected components and the edge original image.  Figure 8 shows an obtained edge image in lane 2.

(6) Broken edges sometimes appear even in edge images obtained by process (5). To remedy this problem, processes (1)–(5) are repeated for five continuous images. Logical operation OR is done among five edge images obtained by process (5), and an edge image is obtained.

(7) The edges of left and right sides of vehicles in the edge image obtained by process (6) are detected by horizontal scan, and the outer sides of vehicle's edges are remained. One-dimensional median filter of length 7 along the edges is employed to correct completely broken edges. The tires' thermal energy reflection areas appear close to the tires. We choose the side in which the area close to tires can be seen from both sides of vehicles. Therefore, the edges of right sides of vehicles are only remained to detect tires' thermal energy reflection areas in lanes 1-2, and those of left sides are only remained in lanes 3-4. Figure 9 shows the detected edges of left and right sides of vehicles.

(8) Histogram equalization is employed for the third original image. To detect the edges of vehicles, we use five continuous images. On the other hand, target of the vehicle detection is third image. In first image processing, we use from frame 1 to frame 5, and target of the vehicle detection is frame 3. In second image processing, we use from frame 2 to frame 6, and target of the vehicle detection is frame 4. In this way, the image processing is continued.

(9) Gamma transformation with *γ* is employed for the image after histogram equalization. Traffic image we used has four lanes. The image has four ROIs (Regions of Interest) to detect vehicles on four lanes. The four vertices of the rectangle measurement area (ROI) on each lane can be freely determined with mouse clicks in accordance with the road shape.  Figure 10 shows an example of four ROIs which are indicated in green lines. Traffic volume and occupancy affect warming up degrees on lanes. The mean of temperature on each lane is different because traffic volume and occupancy in each lane are different. In order to emphasize effectively the tires' thermal energy reflection areas from the road surface, the optimum *γ* value of gamma transformation for each lane is not same. This algorithm chooses 0.1, 0.2, 0.3, or 0.4 as *γ* value for each lane. The process of choosing *γ* value for each lane is described below. Four images are obtained after gamma transformation with four different *γ* values. We call the four images as “IMG-A” in this section. Pixel values in a narrow area near the left or right edge are extracted from IMG-A. The distance between the *x*-coordinate of the narrow area and that of edge is shown in Figure 11. The distance depends on *y*-coordinate. The distance between points A and B is [0.02*y* + 1] and that between points A and C is [0.07*y* + 2]. These geometric parameters [0.02*y* + 1] and [0.07*y* + 2] are affected by the camera angle, the camera height, and the road shape. We decide these parameters by an image processing experiment in advance. The means of pixel values in the narrow areas are calculated. We assume that high pixel values are over 80% of the means, and pixel values except for high pixel values are low pixel values. This algorithm chooses the *γ* value when the ratio of the number of pixels with high pixel values to that of pixels with low pixel values is closest to 0.5. Processes (1)–(9) are repeated 60 times to decide the final optimum *γ* value for each lane. Most selected *γ* value for each lane in 60 times is decided as the final optimum *γ* value. Vehicle detection is not done while this procedure is continued. Therefore, this procedure is usually employed when traffic lights are red.  Figure 12 shows an image in lane 2 after final optimum gamma transformation.

(10) The pixel values are extracted from IMG-A of final optimum *γ* value. The mean of extracted pixel values is calculated. In the calculation, pixel value 0 (black pixel) except for vehicles is not included. The pixel values less than the mean is converted to 0. The pixel values are smoothed by a one-dimensional median filter. The filter size depends on *y*-coordinate and is decided by [0.05*y* + 3]. The pixel values after the median filtering are added to the spatiotemporal image. We call the spatiotemporal image as “ST-IMG” in this section. Thus, ST-IMG is updated.  Figure 13 shows a ST-IMG. ST-IMG is obtained for each lane. In Figure 13, *y*-axis shows *y*-coordinate along detection areas shown in Figure 11, *t*-axis shows elapsed time, and a band with high pixel values shows the tires' thermal energy reflection area of a vehicle. Although method 2 does not use directly the time series of pixel values in detection area to detect vehicles, the tires' thermal energy reflection area of a vehicle appears as the track of each vehicle. Therefore, we can find that the information of tires' thermal energy reflection is effective to detect vehicles from Figure 13.

(11) Each remaining zonal region (high pixel values) in ST-IMG is detected as a vehicle. The location of vehicle detection is shown by a rectangle.  Figure 14 shows the position of the rectangle. The ratio of width to height of a rectangle which shows a detected vehicle is 3 to 2. This ratio is used in method 1 and is the same as the ratio of the positive and negative sample images used in machine learning to construct the multistage cascade of classifiers of Viola-Jones detector. Each vehicle speed is classified based on the ratio of the area of a moving vehicle in a rectangle. Each area of a moving vehicle is detected by spatiotemporal image processing described in Section 2.1. The ratio of a rectangle affects the classification of movement. Therefore, the ratios of method 1 and method 2 are equal. In method 1 [8], we used the smallest rectangle size of 12 × 8 pixels for a detection target area. Therefore, each positive sample image is resized to the 12 × 8 pixels for the training. When computing the regression equation, we fixed the intercept value as 96.0 (12 × 8) which is the minimum area of detection target. We have done a regression analysis between *y*-coordinates and the sizes of the detection target areas (*S*) and obtained the regression equation:

(3) $$\begin{array}{c} S=15.429y+96.0. \end{array}$$

The area of a rectangle in method 2 is calculated by the regression equation (3).

(12) In order to increase vehicle detection accuracy, the following two correction procedures are applied to the vehicle detection result. If the width of a remaining zonal region in ST-IMG is less than [0.08*y*], the region is considered as a noise and is excluded from detected vehicles. The area of a rectangle which shows a vehicle is bigger as the vehicle comes nearer to an infrared thermal camera. So the ratio of the rectangle's area of a vehicle to that of the vehicle just behind it should be over 1.0. We assume that the ratio on lanes 2 and 3 is 1.45 and that on lanes 1 and 4 is 1.35. These geometric parameters 1.45 and 1.35 are affected by the camera angle, the camera height, and the road shape. We decide these parameters by an image processing experiment in advance. If the rectangle's area of vehicle is less than the ratios, the rectangle is erased. This correction procedure erases the vehicle at the front when the distance between two continuous vehicles is too short. This situation is regarded as a double count for a vehicle. Method 2 repeats the processes from (1) to (12).

### 3.3. Vehicle Detection Experiments

We have done the experiments using three video clips of thermal images taken in February for which the vehicle detection accuracies of method 1 are low. We have assumed in the experiments that *k*, *T*_*L*_, and *T*_*H*_ are 2.0, 40, and 50, respectively.

**Figure 15:**
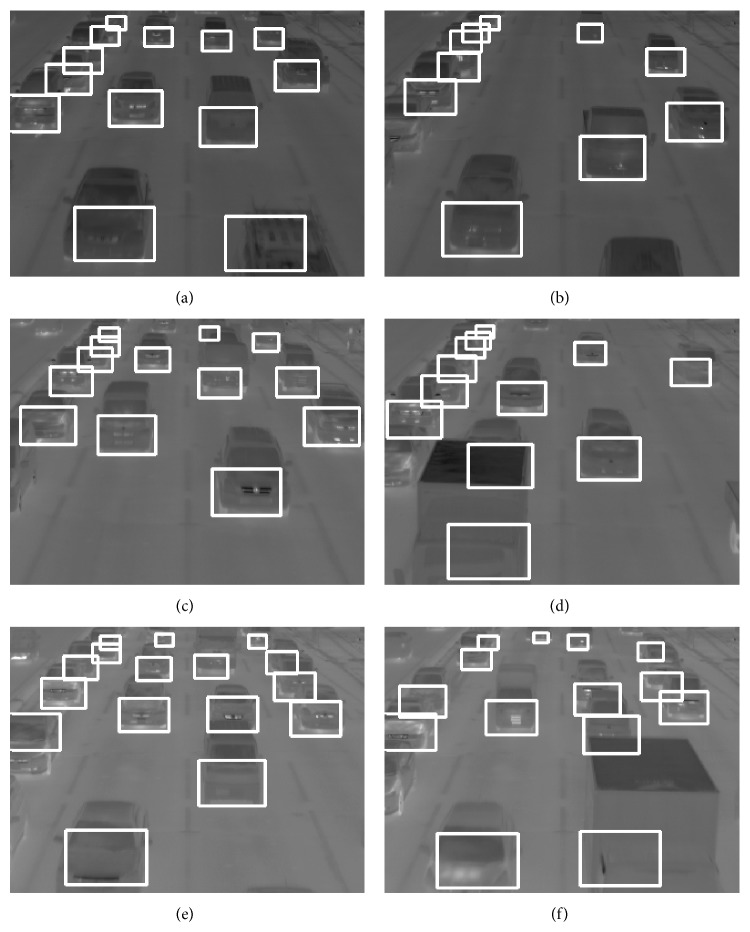
Six examples in detection results for the three video clips of thermal images.

Figure 15 shows six examples in detection results for the three video clips.  Table 2 shows vehicle detection results using the three video clips taken in February.

Method 2 detects the outwardly extended thermal reflection areas on a road surface from tires of vehicles. The thermal reflection area of an occluded vehicle is also spread on a road surface from the tires and can be detected. Therefore, occlusion robust vehicle detection is performed. The vehicle behind the truck on lane 3 in Figure 15(d) can be detected. The trucks in Figures 15(d) and 15(f) can also be detected. However, in rare cases, a truck with a very long length causes misdetection. The three video clips of thermal images using the experiments contain high traffic volumes, and the occlusions of vehicles are seen frequently as shown in Figure 15. Even with those situations, vehicle detection accuracy of method 2 is over 90%.

### 3.4. The Combination of Method 1 and Method 2

Method 2 is applied when the vehicle detection accuracy is reduced by method 1. The spatiotemporal image processing described in Section 2.1 is employed in method 1 and method 2 and can extract vehicle movement areas. A vehicle must exist within a vehicle movement area. First, method 1 is used to detect vehicles within vehicle movement areas. Method 1 sometimes cannot work in winter season depending on outdoor temperature. If method 1 cannot detect the vehicles within vehicle movement areas, method 1 tries again to detect the vehicles by inverting the pixel values of the original image. If method 1 cannot work for the detection of the vehicles within vehicle movement areas, the vehicle detection method is switched from method 1 to method 2.

We compared method 1 and method 2 with the two state-of-the-art algorithms [11, 12] and described the shortcomings of the two algorithms [11, 12] as follows.

1. In the experiments, images of heavy traffic were not used.
2. The number of images used in the experiments and the robustness for the differences of environmental conditions were not shown specifically.

Moreover, the two algorithms [11, 12] do not aim at road traffic flow surveillance.

As far as we have searched, there is not a robust vehicle detection method which can be used in road traffic flow surveillance except for method 1 and method 2.

## 4. An Automatic Traffic Flow Surveillance

We can realize an automatic traffic flow surveillance using the information obtained by method 1 and method 2 (henceforth referred to as “our surveillance”). Our surveillance targets inflow traffic at an intersection as shown in Figures 4(a)–4(c) and Figures 15(a)–15(f). We obtain the information of vehicle positions and their movements classified into three categories: stopped vehicles, slow moving vehicles, and fast moving vehicles in each lane by using method 1 and method 2.

**Figure 16:**
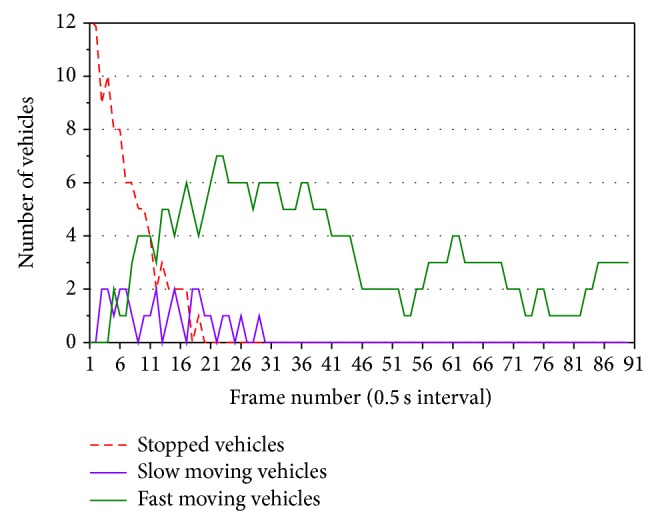
Fluctuations of the number of vehicles in two center-lanes.

Figure 16 shows fluctuations of the numbers of vehicles in classified three categories of movements in lanes 2 and 3. The numbers of vehicles in each category in the two lanes are added. We started measuring the numbers of vehicles at the red phase. So, vehicle queues are extended in measurement area, and many vehicles are stopped. After starting the green phase, vehicles start one after another, and the number of running vehicles is increased. As shown in Figure 16, all vehicles maintain high speeds in the green phase. This shows that traffic is uncongested flow. Our surveillance can also estimate traffic volume in measurement area without time delay. This is effective to control optimally traffic signal lights. We have measured the number of vehicles in classified three categories for uncongested flow using several video clips of thermal images and obtained similar patterns of the fluctuations.

We have done experiments to measure the image processing speed. The main specifications of the personal computer and the software used in the experiments are as follows.

1. CPU: Intel Core i7-2600.
2. RAM: 16.0 GB.
3. OS: Windows 7 64-bit Professional.
4. Programing language: Visual C++.
5. Library: OpenCV.

Experimental results showed that the mean of image processing time per one frame was 0.188 s. In practical applications, hardware implementation like FPGA of the image processing algorithm which is performed by the software described above is desirable to increase the image processing speed.

All vehicles maintain high speeds in uncongested flow. Therefore, if the number of slow moving vehicles and stopped vehicles increases suddenly in the green phase, occurrence of a traffic incident is estimated. We can detect a traffic incident by counting simply the numbers of vehicles classified into three movement categories. We judge that a traffic incident occurred when the following inequality is satisfied:

(4) $$\begin{array}{c} n_{\text{stop}}+n_{\text{slow}}\geq n_{\text{fast}}, \end{array}$$

where *n*_stop_ is the number of stopped vehicles, *n*_slow_ is the number of slow moving vehicles, and *n*_fast_ is the number of fast moving vehicles.

**Figure 17:**
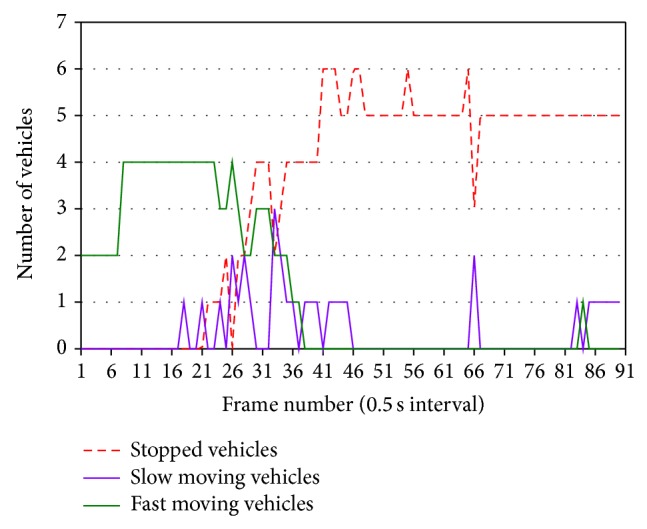
Fluctuations of the number of vehicles in lane 2.

**Figure 18:**
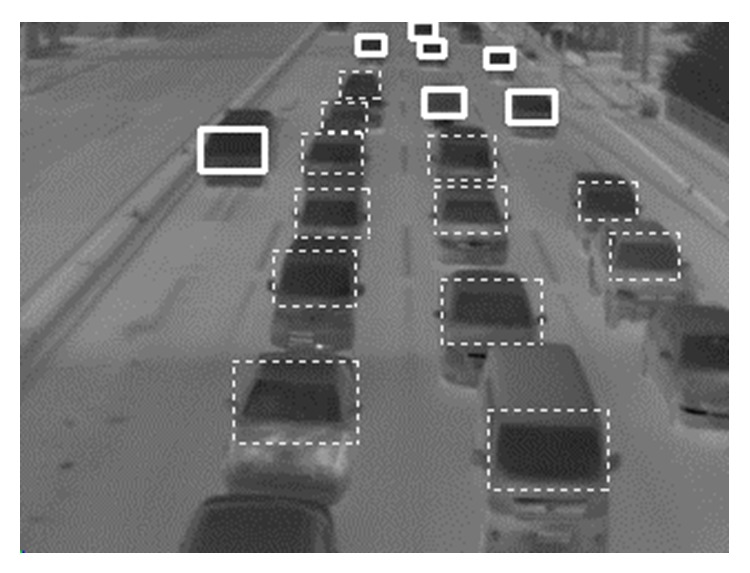
The image of frame number 30 in Figure 17.

Figure 17 shows that traffic jam in lane 2 increases in the middle of the green phase, due to downstream jam. In Figure 17, (4) is satisfied on and after frame number 27.  Figure 18 shows the image of frame number 30 in Figure 17.

Method 1 and method 2 provide vehicle positions and their movements. Lane changes cannot be detected strictly because method 1 and method 2 do not have a tracking function. However, increase or decrease of the number of vehicles on each lane caused by lane changes can be detected. The absence of a tracking function is not problem for our surveillance.

We can also detect other traffic incidents. If a vehicle stops suddenly in the green phase, the stopping vehicle is detected and it is estimated as a broken down vehicle or illegal parking. If such an event occurs in lane 1, the possibility of illegal parking is higher. If two or more vehicles stop suddenly in the green phase, the stopping vehicles are detected and they are estimated as a traffic accident.

## 5. Conclusions

First, we have explained briefly method 1 which detects vehicle positions and their movements by using thermal images obtained with an infrared thermal camera. Method 1 uses the windshield and its surroundings as the target of pattern recognition using the Viola-Jones detector. In a condition in winter, the vehicle detection accuracy decreases because the temperatures of many windshields approximate those of the exteriors of the windshields. To remedy this problem, we have proposed method 2 without relying on this pattern recognition. Method 2 detects vehicles based on distinguishing the tires' thermal energy reflection areas on a road surface from other areas. We have done the experiments using three video clips of thermal images for which the vehicle detection accuracies of method 1 are low. Method 2 detects 3,084 vehicles (92.1%) out of 3,348 vehicles, and the number of false detection is 154 in total. Occlusion robust vehicle detection is performed because method 2 uses the thermal information of the outside of vehicles. Three video clips of thermal images using the experiments contain high traffic volumes, and the occlusions of vehicles are seen frequently. Even with those situations, vehicle detection accuracy of method 2 is over 90%. Therefore, by combining method 1 and method 2, high vehicle detection accuracies are maintained under various environmental conditions.

We have realized our surveillance using the information obtained by method 1 and method 2. It is possible to distinguish uncongested flow and congested flow in each lane. We can also detect traffic incidents such as traffic accidents, broken down vehicles, and illegal parking under various environmental conditions. By using our surveillance, we also expect to realize optimal traffic signal control.

As a future subject, we will perform further experiments to investigate the characteristics of method 2 in more detail.

## Figures and Tables

**Table 1:** Vehicle detection results using three video clips.

Month	June	August	October	Total of three months: June, August, and October
Number of used images	64	64	64	192
Intervals (s)	1	1	1	1
Number of vehicles counted by sight	483	596	381	1,460
Number of vehicles detected by method 1	460	574	370	1,404
Detection accuracy (%)	95.2	96.3	97.1	96.2
Number of false detection	9	8	18	35

**Table 2:** Vehicle detection results using three video clips taken in February.

Number of used images	287

Intervals (s)	1

Number of vehicles counted by sight	3,348

Number of vehicles detected by method 2	3,084

Detection accuracy (%)	92.1

Number of false detection	154
